# Effect of Centring Rings on Number of Retakes and Cone Cuts in Intraoral Imaging Performed by Dental Students: A Randomized Case–Control Study

**DOI:** 10.1111/eje.70022

**Published:** 2025-07-22

**Authors:** Louise Hauge Matzen, Lars Schropp

**Affiliations:** ^1^ Oral Radiology, Department of Dentistry and Oral Health Aarhus University Aarhus Denmark

**Keywords:** cone cuts, dental students, intraoral imaging, retakes

## Abstract

**Aim:**

To assess whether the use of centring rings for intraoral radiographic imaging reduces the number of retakes or the frequency and size of cone cuts in intraoral images performed by dental students.

**Material and Methods:**

Seventy‐three dental students were randomly allocated to either a test or control group before training intraoral imaging on a phantom. Both groups used phosphor plate holders for periapical imaging of anterior and posterior teeth and for bitewings. Additionally, the test group used dedicated centring rings mounted to the holder (Kerr, Hawe, USA). After phantom training, the students performed intraoral imaging in 127 patients. Before patient examination, the number of planned periapical images and bitewings was recorded. After patient examination, the percentage of retakes was calculated in addition to the frequency and size of cone cuts. Differences between the two groups were tested with the Mann–Whitney *U* test (percentage of retakes and size of cone cuts) and chi‐squared test (frequency of cone cuts).

**Results:**

The test group performed 920 intraoral images and the control 835. The percentage of bitewing retakes was significantly lower in the test group (*p* = 0.014), whereas there was no difference in periapical retakes (*p* = 0.37) between the groups. The frequency of cone cuts was significantly lower in the test group for both periapical images and bitewings (*p* < 0.001). The size of cone cuts was lower in the test group, although this was not significant (*p* > 0.20).

**Conclusion:**

In general, it seems beneficial to use centring rings for dental students when learning to perform intraoral imaging since the percentage of retakes as well as the frequency and size of cone cuts were reduced.

## 1 Introduction

Performing intraoral radiographic examinations intends to obtain sufficient images to be used for diagnostics and treatment planning. Using a receptor holder for the radiographic examination has been shown to result in reliable images that can be repeated compared to supporting the receptor with a finger [1].

During the radiographic examination, retakes should be avoided since in every radiographic examination the patient is exposed to radiation. A receptor holder aims to stabilise the receptor in the mouth behind and parallel to the long axis of the teeth, and moreover it serves as an aiming device so the receptor is sufficiently irradiated. Using holders, the number of retakes should therefore theoretically be reduced.

The diagnostic quality of an image can be impaired with the presence of cone cuts, which in turn can also increase the need for a retake if the tooth and related structures in question are not fully displayed in the image. In an older study from 1979, cone cutting was seen in 20.8% of the images performed [2], while a recent study from 2023 showed some degree of cone cut in 21% of radiographs taken using a rectangular collimator [3]. Cone cuts have been reported to occur more often when an intraoral radiographic examination is performed with a rectangular collimator compared to a cylindrical one [4]. To guide the positioning during the intraoral radiographic examination, it has also been suggested to use centring devices to avoid cone cuts [5, 6, 7] although this can be challenging as well.

Performing intraoral radiographic images with sufficient quality is part of the curriculum during dental education. It is of utmost importance that students are taught this in a way so they can achieve adequate images for diagnosis with as few retakes as possible. There are no strict guidelines on how to structure a dental curriculum with regard to intraoral imaging training, and therefore studies on this topic are relevant. In a retrospective cross‐sectional study from 2009, 35.9% of the periapical images performed by undergraduate students were judged unacceptable [8]. In another retrospective cross‐sectional study from 2017, a similar repeat rate for intraoral digital periapical imaging performed by undergraduate students was reported (34.4%) and the repeat rate was 15.1% for bitewings [9]. It is unclear whether a holder was used for the periapical images.

One study from 1996 examined differences in the number of retakes and cone cuts between two groups of dental students [10]. Both groups consisted of 13 dental students who performed a full‐mouth periapical radiographic examination with a cylindric collimator of 7 cm and conventional films. Both groups used film holders. One group used a holder with a round aiming device, and the other group used an aiming plate with a central collimated rectangular opening. In total, 78 patients were examined. There was no significant difference between the two groups in the number of retakes. On the other hand, there was more cone cutting and improper film positioning in the group that used the rectangular aiming device. In a recent study, it was concluded that the use of a training kit including dental cast models, film holder and centring rings for cylindric collimation was beneficial for intraoral imaging simulation before further patient examination conducted by dental undergraduates [11].

At the dental schools in Denmark, a rectangular collimator has been used for decades for intraoral imaging to reduce radiation to the patient. The Danish authorities set by law that from 2023, a collimator only with a size of 4 × 5 cm is permitted to use in the dental clinics [12].

The aim of this study was to evaluate whether the use of centring rings for intraoral imaging reduces the number of retakes and the size and frequency of cone cuts in a learning environment of dental students.

## 2 Materials and Methods

This case–control study was conducted during spring 2023 and involved 73 second‐year dental students from the Department of Dentistry and Oral Health, Aarhus University, Denmark. The students were informed about the present research project, and all of them accepted to participate. The participants were anonymised during data collection and data treatment as only the group affiliation was noted. All students received theoretical radiology teaching on intraoral imaging, including lectures and videos on radiation, radiation protection, dental apparatus and equipment and projection geometry before starting up the practical radiographic training.

The study included data from intraoral images performed at Section of Oral Radiology and Endodontics of patients who were referred by the student clinic. The images should serve as the basis for the overall treatment planning at the dental school. This type of study was not considered a “health research study” and therefore, according to the Danish guidelines, ethical approval for performing the study was not needed.

### 2.1 Case and Control Group

**FIGURE 1:**
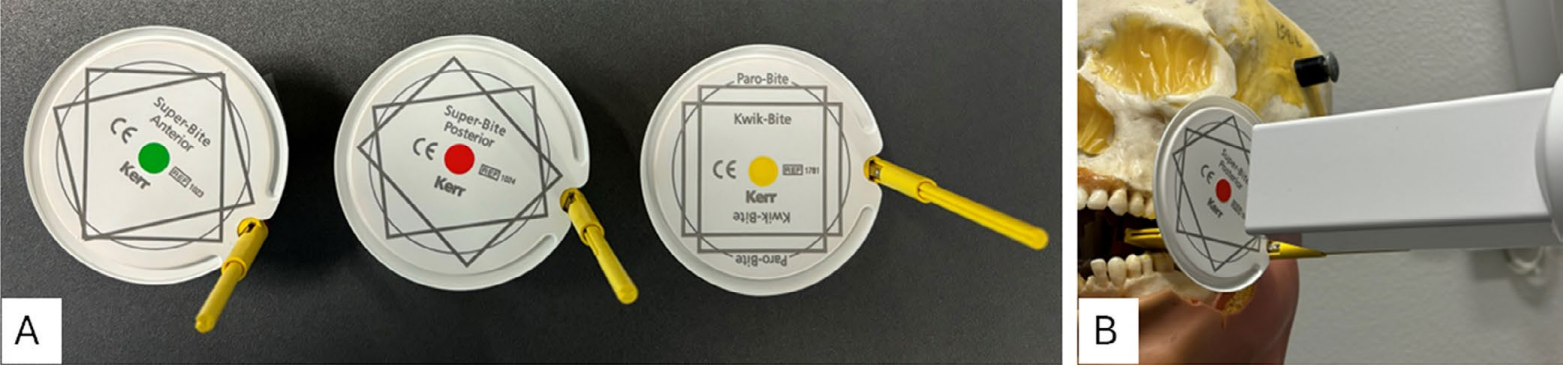
(A) Receptor holders with centring rings. (B) Use of a posterior centring ring on a phantom.

The students were randomly allocated to either a test group (37 students) or a control group (36 students). Both groups were taught to use receptor holders (Super‐Bite; Kerr, Hawe, Orange, CA, USA) for periapical imaging of anterior and posterior teeth and for bitewing examinations. Additionally, the test group was taught to use the dedicated centring rings mounted to the holders (Super‐Bite with ring; Kerr, Hawe, Orange, CA, USA) (Figure 1). Before radiographic examination of patients, the students practised on a phantom head performing a full‐mouth intraoral examination with periapical images of all teeth supplemented with bitewings. If it was judged that a student was not capable of taking the radiographs sufficiently, he/she was asked to return another day for additional training on the phantom head. After approval by the clinical instructor, the students were allowed to proceed with radiographic examination in patients. For practical reasons, different instructors were involved in the teaching, but only one at a time. All the instructors were experienced in the specific task and were calibrated in the evaluation of the radiographs specifically. The students performed intraoral radiographic examinations on patients at two occasions with two‐week interval.

### 2.2 Radiographic Examination

The intraoral images were acquired with a MINRAY/FOKUS X‐ray unit (KaVo, Biberach/Riß, Germany) and by using one of two phosphor plate systems, either VistaScan (Dürr Dental, Stuttgart‐Feuerbach, Germany) or Digora Optime (KaVo, Biberach/Riß, Germany), and an image receptor size 0 or 2 was used according to the guidelines at the radiology clinic. Size 0 was used in the vertical position for incisors and canines, and Size 2 was used in the horizontal position for premolars and molars, as well as for the bitewing images. A rectangular collimator of 4 × 5 cm was used according to Danish legislation. If a patient did not comply and the examination based on the referral could not be conducted, the patient was excluded from the study.

### 2.3 Image Assessment and Approval

**FIGURE 2:**
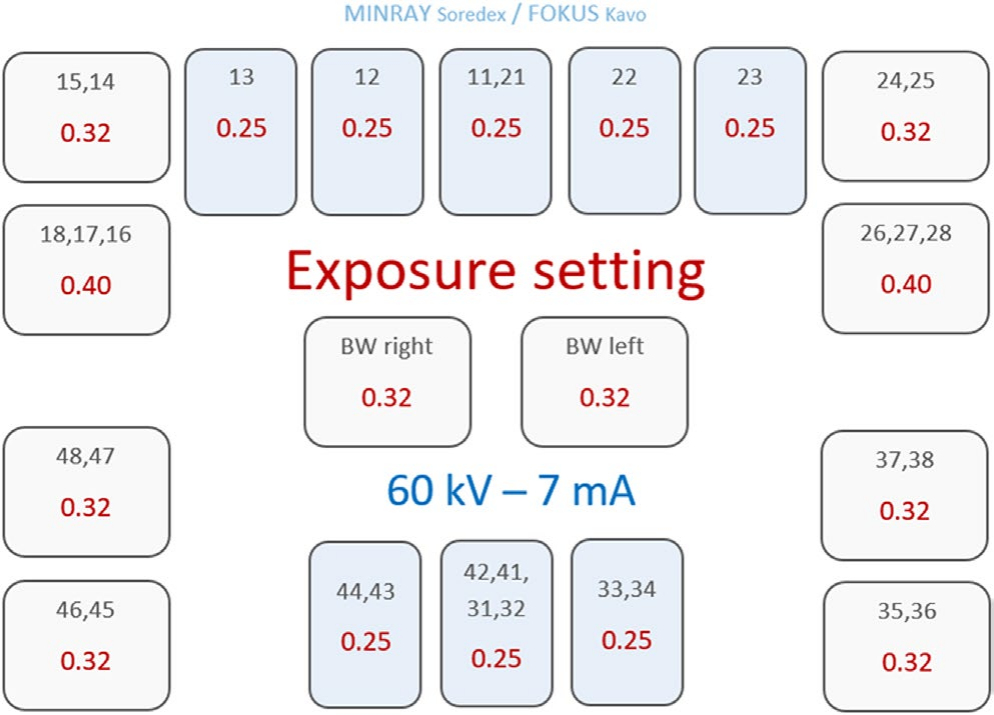
Overview of a full mouth radiographic examination performed at the department including receptor size and exposure parameters. Blue squares indicate that a Size 0 receptor should be used, and white squares indicate that a Size 2 receptor should be used. Exposure times are specified in seconds.

**FIGURE 3:**
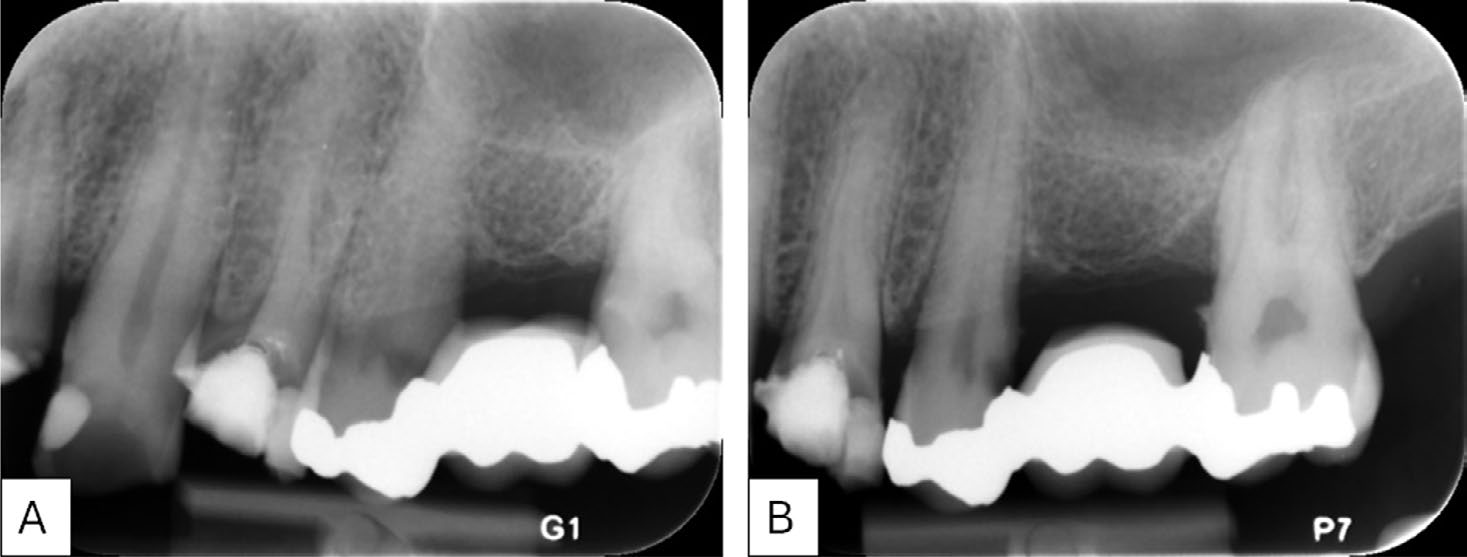
A retake was needed (B) as there was overlap between the teeth and furthermore, the periapical area was not sufficiently displayed (A).

Figure 2 shows an overview of intraoral imaging at the radiology clinic. Based on the referral, the students performed the corresponding images (Figure 2). After each examination, the clinical instructor decided if the image was sufficient or whether a retake must be performed. A sufficient periapical image displays the entire tooth/teeth of interest and 2–3 mm of surrounding periapical bone must also be displayed. For periapical images and bitewings, the proximal sites must be separated with no or minimal overlap. If this was not obtained, a retake was performed. The instructor focused on keeping the radiation exposure to the patients as low as possible, adhering to the ALADA principle in this decision. An example of a retake is shown in Figure 3. Retakes due to technical/scanning errors were excluded from the study analyses.

**FIGURE 4:**
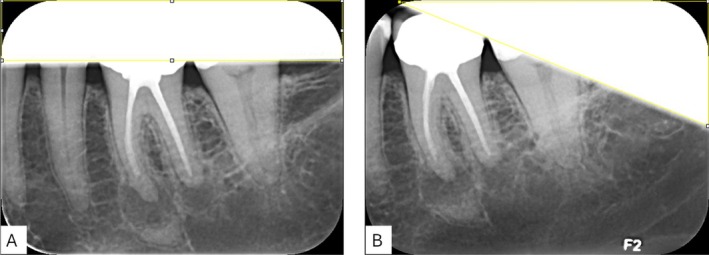
Two examples of how the size of a cone cut was measured in relation to the size of the total image. In image (A) the cone cut constitutes 23.8% of the total image, and in (B) the cone cut constitutes 22.0% of the image.

If a cone cut was present in the intraoral image, the image was imported into an image viewing software (ImageJ, National Institutes of Health, Maryland, USA) for measuring the size of the cone cut area as well as the size of the total image as illustrated in Figure 4.

### 2.4 Data Treatment

For each patient, the number of periapical images and bitewings needed to fulfil the referral was recorded before the radiographic examination. After the radiographic examination, the actual number of periapical images and bitewings, respectively, performed was recorded, and the number of retakes was calculated in percentage. As an example, a patient referred for radiographic examination of 17, 16, 11, 21, 24, 43, 31 and 35 needed six images according to the intraoral radiographic examination guideline (Figure 2). If the students in this patient performed a retake of 17 and 35, obtaining eight images in total, then the retake percentage was 33.3%. The difference in the percentage of retakes between the test and control group was tested by Mann–Whitney *U* test.

Frequencies of cone cuts—both for the first radiographs and the retakes—were calculated, and the size of cone cuts was expressed as a percentage of the total area of the image. Mann–Whitney *U* statistics tested the mean difference in the size of cone cuts between the test and control groups, and chi‐squared test tested whether there was a difference in the frequency of cone cuts between the two groups.

## 3 Results

One hundred twenty‐seven patients underwent intraoral radiographic examination during the study period. In total, the test group performed 920 intraoral images in 61 patients, and the control group performed 835 intraoral images in 66 patients. Table 1 shows the distribution of images.

**TABLE 1:** Number and distribution of intraoral images.

	Without centring (control)	With centring ring (test)
Periapical images		
Planned	407	465
Performed	611	753
Retakes	204	288
Cone cuts	266	171
Bitewings		
Planned	107	98
Performed	224	167
Retakes	117	69
Cone cuts	131	50

The number of periapical retakes in the test group was 288 (62% more images performed than planned) and 204 (50% more images performed than planned) in the control group. The difference between the groups was not statistically significant (*p* = 0.37). On the other hand, there was a statistically significant difference in the number of bitewing retakes (*p* = 0.014) between the two groups, with 69 retakes (70% more images performed than planned) in the test group and 117 (109% more images performed than planned) in the control group.

In the test group, there were 23% of the performed periapical images with cone cuts and 30% of the performed bitewing images with cone cuts, while 44% of the performed periapical images in the control group were associated with a cone cut and 58% of the bitewing images. The frequency of cone cuts was significantly lower in the test group for both periapical and bitewing images (*p* < 0.001).

The size of cone cuts in the periapical images ranged from 0.5% to 69.8% for the test group compared to 0.6%–94.6% for the control group. For bitewing images, the size of cone cuts ranged from 0.3% to 36.0% for the test group and 0.5%–44.2% for the control group. The mean size of the cone cuts (Table 2) was thus lower in the test group compared to the control for both periapical and bitewing images, but the differences were not statistically significant (*p* = 0.36 and *p* = 0.20, respectively).

**TABLE 2:** Mean size of a cone cut in percentage of the size of the total image.

	Without centring ring (control)	With centring ring (test)
Periapical images	10.9%	9.9%
Bitewings	10.3%	8.7%

## 4 Discussion

The overall aim of performing intraoral radiographic imaging is to obtain a sufficient image with good image quality displaying the tooth or teeth in question in the correct dimensions and with no overlap between teeth, and including relevant surrounding bone and anatomical structures without the need for retakes. This is part of the dental curriculum to teach dental students in this respect. In the present study, the performance of second‐year dental students training in intraoral imaging was assessed. The students were allocated by randomisation into either a test or control group to examine the impact on cone cuts and retakes, where the test group used a centring ring attached to the receptor holder, and the control group only used the receptor holder.

It can be discussed how an optimal intraoral radiology education programme should be organised. In the present study, the students had theoretical teaching about intraoral imaging including lectures on dental equipment, radiation physics and projection geometry. Thereafter, they trained on phantom heads before performing radiographic examinations on patients. The students learned to use the paralleling technique and a receptor holder and the radiographic examinations were performed with a rectangular collimator. The results of the present study revealed that the use of centring rings reduced the number of bitewing retakes significantly and moreover for both periapical images and bitewings, the frequency of cone cuts was significantly lower and the size was smaller when using a centring ring. Thus, it seems that there is a benefit of using a centring ring for dental students without experience in intraoral imaging. It is noteworthy, however, that the number of retakes for the periapical radiographs was not reduced in the test group, which may indicate that the use of the centring ring possibly complicates the imaging procedure for unexperienced users (more things to control), which in turn may generate other errors than those related to cone cuts, for example, exposure on the wrong side of the phosphor plate, incorrect placement of the plate in the mouth, etc. A previous study evaluated the use of a simulation training kit for intraoral imaging [11]. Ten students, who beforehand had radiographic images rejected in their clinical training, were included in the study. The students were divided into a test and a control group. Both groups were exposed to demonstration videos on correct utilisation of film and holder and placement in the mouth. In addition, the test group trained with a training kit comprising dental cast models and four sets of film holders with an aiming device intended for a cylindric collimator. There was no significant difference between the two groups with regard to rejected images. On the other hand, for the test group there were significantly fewer retakes after training with the training kit than before the test period. This was not the case for the control group only seeing the demonstration videos. Therefore, simulation seems beneficial. In another study, the students were taught to use the bisecting angle technique [13]. From the results of that study, it was decided to re‐evaluate the educational programme because of too many imaging errors. The included students were fourth‐year students who already had passed two practical training courses in intraoral imaging.

The educational level of dental students differs among studies on the subject. Based on the results from two studies, it was concluded that the frequency of retakes decreased with increasing level of experience [14, 15], and in one study it was stated that a dental student needs to perform 20–25 full mouth intraoral examinations before obtaining a sufficient level of experience [15]. On the other hand, in a recent study, there was no difference among third‐, fourth‐ and fifth‐year dental students in the number of accepted and rejected intraoral images [16]. In that study, the rejection rate was in general low for periapical images (5.1%) and bitewings (9.8%), respectively [16]. In another study, 35.94% of periapical images performed by undergraduate students were unacceptable [8]. In the present study, the frequency of periapical retakes was 62% for the test group and 50% for the control group and for bitewings the frequency of retakes was 70% for the test group and 109% for the control group. In general, the numbers of retakes/rejections from the present study were higher compared to those in the literature. It is not clearly stated in the studies why images were rejected and a retake was performed. In the present study, a rectangular collimator was used whereas they used a cylindrical collimator in other studies. It is known that more cone cuts are present in images performed with a rectangular collimation [4], and this together with the fact that the students in the present study were totally inexperienced may explain some degree of difference in the reported results. A recent study has compared round and rectangular collimators in a dental school radiology clinic and demonstrated that although the number of retakes increased with the transition from round to rectangular collimators, a decrease in effective dose was observed [17]. The study also showed that the number of retakes decreased as students gained more experience and practice with rectangular collimation.

There is no threshold for an acceptable retake rate but naturally, it must be as low as possible. Different reasons for imaging errors besides processing errors have been reported such as cone cutting, incorrect vertical angulation, incorrect horizontal angulation and incorrect receptor placement. Several studies have reported that one of the most common reasons for performing a retake was cone cutting [2, 8, 10, 15, 16, 18]. A study from 2022 revealed that most retakes were due to placement errors, defined as missing contact points, missing apices of teeth, or a missing area of interest [17]. In an audit by Patankar et al. [7], they ended up with seven recommendations for intraoral imaging and one of them was to use receptor positioning devices using the long cone technique facilitating rectangular collimation whenever possible. In the present study, a positioning device was used in the test group, which resulted in fewer cone cuts and if cone cuts were present, they were smaller than the cone cuts in the images from the control group, so our results support the recommendation by Patankar et al. [7].

In the present study, the number of retakes and the frequency and size of cone cuts were examined in relation to the use of a receptor holder system including a centring ring. This study did not assess other reasons for imaging errors or if specific anatomic regions were more prone to errors. Other studies have assessed the rejected images in relation to the anatomic location. In one study, it was found that there was a significant correlation between errors and anatomic location and most errors were observed in the maxillary molar area [8]. Another study from an Indian dental school had a repeat rate of 7.1% with 57% of them in the upper jaw and 42% in the lower jaw and 1% bitewing retakes [18]. In that dental school, they used the bisecting angle technique, film and a rectangular collimator. Moreover, they reported that most errors were due to incorrect positioning resulting in cone cuts. A recent study assessing more than 50 000 images found some degree of cone cut in 21% of the radiographs, while the diagnostic value was unaffected in 18% of the radiographs with cone cut, and only 3% of the radiographs were deemed diagnostically unusable due to cone cut [3]. Most cone cuts were in the premolar and molar areas, while it was least likely to be diagnostically unusable in the front area.

Based on the results of the present study, the students in the control group were introduced to and taught in using the centring rings in the following semesters. Furthermore, the use of centring rings has now been implemented in the practical sessions in radiology at the dental school in Aarhus.

In conclusion, it seems beneficial to use a centring ring attached to the receptor holder when teaching second‐year dental students intraoral imaging using the paralleling technique and a rectangular collimator. In general, the dental schools should emphasise the importance of practical intraoral radiology teaching to reduce the number of insufficient images and retakes.

## Conflicts of Interest

The authors declare no conflicts of interest.

## Data Availability

The data that support the findings of this study are available from the corresponding author upon reasonable request.
